# Comparing the content of participation instruments using the International Classification of Functioning, Disability and Health

**DOI:** 10.1186/1477-7525-7-93

**Published:** 2009-11-13

**Authors:** Vanessa K Noonan, Jacek A Kopec, Luc Noreau, Joel Singer, Anna Chan, Louise C Mâsse, Marcel F Dvorak

**Affiliations:** 1 Division of Spine, Department of Orthopaedics, University of British Columbia, Vancouver, BC, Canada; 2 School of Population and Public Health, University of British Columbia, Vancouver, BC, Canada; 3 Arthritis Research Centre of Canada, Vancouver, BC, Canada; 4 Rehabilitation Department, Laval University, Québec City, QC, Canada; 5 Centre for Interdisciplinary Research in Rehabilitation and Social Integration, Québec City, QC, Canada; 6 Canadian HIV Trials Network, Vancouver, BC, Canada; 7 Department of Pediatrics, University of British Columbia, Vancouver, BC, Canada

## Abstract

**Background:**

The concept of participation is recognized as an important rehabilitation outcome and instruments have been developed to measure participation using the International Classification of Functioning, Disability and Health (ICF). To date, few studies have examined the content of these instruments to determine how participation has been operationalized. The purpose of this study was to compare the content of participation instruments using the ICF classification.

**Methods:**

A systematic literature search was conducted to identify instruments that assess participation according to the ICF. Instruments were considered to assess participation and were included if the domains contain content from a minimum of three ICF chapters ranging from *Chapter 3 Communication *to *Chapter 9 Community, social and civic life *in the activities and participation component. The instrument content was examined by first identifying the meaningful concepts in each question and then linking these concepts to ICF categories. The content analysis included reporting the 1) ICF chapters (domains) covered in the activities and participation component, 2) relevance of the meaningful concepts to the activities and participation component and 3) context in which the activities and participation component categories are evaluated.

**Results:**

Eight instruments were included: Impact on Participation and Autonomy, Keele Assessment of Participation, Participation Survey/Mobility, Participation Measure-Post Acute Care, Participation Objective Participation Subjective, Participation Scale (P-Scale), Rating of Perceived Participation and World Health Organization Disability Assessment Schedule II (WHODAS II). 1351 meaningful concepts were identified in the eight instruments. There are differences among the instruments regarding how participation is operationalized. All the instruments cover six to eight of the nine chapters in the activities and participation component. The P-Scale and WHODAS II have questions which do not contain any meaningful concepts related to the activities and participation component. Differences were also observed in how other ICF components (body functions, environmental factors) and health are operationalized in the instruments.

**Conclusion:**

Linking the meaningful concepts in the participation instruments to the ICF classification provided an objective and comprehensive method for analyzing the content. The content analysis revealed differences in how the concept of participation is operationalized and these differences should be considered when selecting an instrument.

## Background

Participation is cited as central to a person's quality of life and well-being [1]. The reduction of disabilities and improving participation for individuals with disabilities are therefore important goals of rehabilitation [2]. Working for pay, attending school and joining in community activities are all examples of life situations that comprise participation. Participation is defined in the International Classification of Functioning, Disability and Health (ICF) as the 'involvement in a life situation' and participation restrictions are defined as 'problems an individual may experience in the involvement in life situations' [3]. Although the idea of participation is not new, participation as defined in the ICF is a relatively new concept and as a result the conceptualization and measurement of participation continues to evolve [4].

Whiteneck [5] in his critique of the ICF recommended that new instruments operationalizing the concepts in the ICF be developed and tested to assess the relationship among the concepts in the ICF model. Instruments should be pure measures and not contain content from other ICF concepts if the intent is to examine the relationship among the concepts in the ICF model [6]. Furthermore, if instruments are to be used to evaluate treatment effects then the content of the individual questions must be clearly understood since there is a chance of not capturing the effect if multiple outcomes are assessed [6]. It is therefore necessary to identify participation instruments developed using the ICF and then examine the content to determine how the concept of participation has been operationalized and if content pertaining to other concepts is included.

In 2003 Perenboom and Chorus [2] reviewed the literature and examined how existing generic instruments assess participation according to the ICF. These authors concluded that most of the instruments evaluate one or more domains related to participation but none of them measure all the domains [2]. Since Perenboom and Chorus [2] conducted their review, new instruments have been developed using the ICF. A preliminary version of the ICF was published in 1997 and the first version was published in 2001, as a result few of the instruments included in the Perenboom and Chorus [2] review were based on the ICF model. The methodology for linking content of instruments to the ICF classification has been developed [7,8] and this methodology is recommended since it provides a standardized framework for evaluating content [9]. To date, this methodology has been used to compare the content of both generic and disease-specific instruments [9,10]. The purpose of this study was to build on the work by Perenboom and Chorus [2] and examine the content of instruments measuring participation according to the ICF using the published methodology.

## Methods

### Concept of Participation

In the ICF model the concepts of activity and participation are differentiated, but in the classification these concepts are combined and there is a single list of domains covering various actions and life areas. The user is provided with four options on how activity and participation can be considered: 1) divide activity and participation domains and do not allow for any overlap; 2) allow for partial overlap between activity and participation domains; 3) operationalize participation as broad categories within the domains and activity as the more detailed categories, with either partial or no overlap; and 4) allow for complete overlap in the domains considered to be activity and participation [3]. Similarly, in the literature there is no consensus regarding how activity is differentiated from participation [2,5,11-14]. Some have suggested that participation comprises life roles [2] whereas others have used multiple criteria to differentiate these concepts [5].

In this study option number one (described above) was selected to differentiate these two concepts. The following ICF domains (or chapter headings) were considered relevant to the concept of participation: *Communication*; *Mobility*; *Self-care*; *Domestic life*; *Interpersonal interactions and relationships*; *Major life areas*; and *Community, social and civic life *(*Chapters 3 *to *9 *respectively). For the purpose of this study, chapter headings were used instead of interpreting the individual questions according to criteria since it was felt to be more objective. *Chapter 1 Learning and applying knowledge *and *Chapter 2 General tasks and demands *cover content primarily related to the ICF concept of activity, defined as 'execution of a task or action by an individual' [3] and were therefore not included.

### Instruments

A systematic search of seven databases [Medline; CINAHL; EMBASE; HaPI; Psyc (Info, Articles, Books)] was conducted to identify all the instruments that assess participation and were based on the ICIDH-2 or ICF model. The ICIDH-2 was first released in 1997 and so the search included articles published between 1997 and March 2008. Instruments including domains covering a minimum of three chapters in the ICIDH-2 participation dimension, or three chapters from the ICF *Chapters 3 *to *9 *in the activities and participation component, were considered to assess participation. A minimum of three ICIDH-2 participation dimensions or three ICF chapters were required in order to exclude specific instruments (e.g. employment instruments).

Instruments which met this definition of participation were then included if they were designed to assess participation in the community, either self-administered or interview administered, generic in content, developed for adults and published in English. A list of the search terms is provided in the Appendix.

### Linking to the ICF Classification

For each instrument all questions were assigned ICF categories or codes, also known as linking or cross-walking. First the content contained within each of the questions and, if applicable, response options (response scale) were identified using standardized linking rules [8]. This content is referred to as the meaningful concept(s) in the published methodology [8]. The meaningful concept(s) capture all of the ideas or information contained within a question and these concepts are used to select the ICF categories in the classification.

The ICF consists of two parts: functioning and disability and contextual factors. Functioning and disability contains the following components: body structures, body functions, and activity and participation. Contextual factors comprise the background of a person's life and living which interact with the individual and determine their level of functioning [3]. They include environmental and personal factors. Environmental factors include the physical, social and attitudinal environment in which people live [3]. These factors are external to individuals and can have a positive or negative influence on an individual's performance as a member of society, on an individual's capacity to execute actions or tasks, or on an individual's body functions or structures [3]. Personal factors are the particular details of an individual's life and include factors such as gender, age and coping style [3]. A detailed classification of environmental factors was first introduced in the ICF and currently a classification does not exist for personal factors. In addition, the ICF model includes the health condition (disorder or disease) which is classified using the World Health Organization's etiological classification, the International Classification of Diseases-10 (ICD-10) [3].

To determine if contextual factors and health conditions are included in the participation instruments, relevant information stated in the instructions was also used to identify meaningful concepts, which is a modification to the published linking rules. For example, if the instructions state the respondent should consider the impact of his or her health condition or the use of assistive devices when thinking about participating in certain life roles, then 'health conditions' and 'assistive devices' were included as meaningful concepts for each question. The meaningful concepts in the instructions were included for each question since a person should consider the instructions when answering each question and it also ensures the content is comparable among the instruments.

Any terms referring to a time period (e.g. in the past four weeks) and qualifiers such as 'difficulty', 'satisfaction' or 'importance' were not considered to be meaningful concepts. To ensure the meaning of each question was captured, meaningful concepts could be repeated within the instruments; as an example, if an instrument has five to six questions which are related to each aspect of participation (e.g. dressing) then 'dressing' was considered a meaningful concept in each of the six questions to determine how many questions ask about dressing. If examples are used to describe an aspect of participation then all the examples were coded as meaningful concepts and linked to ICF categories. Meaningful concepts were also identified in screening questions since these questions ask about aspects of participation.

The ICF classification was then used to assign ICF categories to the meaningful concepts. In the ICF classification the components are labeled with letters: body structures (s), body functions (b), activity and participation (d), and environmental factors (e). As mentioned previously, personal factors are not specified. Within each component in the ICF, the categories are organized hierarchically and assigned a numeric code. The categories are nested so the chapters also referred to as domains, include all the detailed subcategories. An example demonstrating the coding from the activities and participation component is *d5 Self-care *(chapter/first-level category), *d540 Dressing *(second-level category) and *d5400 Putting on clothes *(third-level category). The ICF classification allows the meaningful concepts to be linked to very detailed categories and the categories can be rounded up to examine coverage in broad aspects of participation.

The meaningful concepts were linked to the most precise ICF category, ranging from the chapter (1 digit code) to the fourth-level (5 digit code). According to the published linking rules [8], the 'other specified' and the 'unspecified' ICF categories should not be used. The meaningful concept was coded as 'not definable' if there was not enough information to select the most precise ICF category and if a meaningful concept was not included in the ICF (e.g. suicide attempts) it was coded as 'not covered' [8]. A meaningful concept was coded as a 'personal factor' if it asks about age or other factors that relate to the background of the person. Meaningful concepts such as health, illness or physical disability were coded as 'health condition'. Examples of the meaningful concepts extracted from the questions and the assigned ICF categories and codes are provided in Table 1. One coder was primarily responsible for determining the meaningful concepts and two coders linked the meaningful concepts in the instruments. The results were compared and the coders discussed the questions where different ICF categories were selected. Another coder was consulted if there were any questions regarding the meaningful concepts, ICF categories or codes and made the final decisions. All the coders were familiar with the ICF and the linking rules [8].

**Table 1:** Examples of linking questions to ICF categories and codes

Question	Meaningful Concept	ICF Category or Code Assigned
During the past 4 weeks, I have moved around in my home, as and when I have wanted to.	moving around in my home	d4600 Moving around within the home
*It does not matter if you require the help of other people or from gadgets and machines*.* (KAP)	*assistance from others *	*e3 Support and relationships *
	*use of gadgets/machines*	*e120 Products and technology for personal indoor and outdoor mobility and transportation*
In the last 30 days how much difficulty did you have in dealing with people you do not know.	dealing with strangers	d730 Relating with strangers
*This questionnaire asks about difficulties due to health conditions*.* (WHODAS II)	*health condition*	*health condition*

Abbreviations:

KAP, Keele Assessment of Participation; WHODAS II, World Health Organization Disability Assessment Schedule II

Notes:

* the text in italics are the instructions for the instrument and the relevant information that was included as meaningful concepts and coded.

### Analysis

First a descriptive analysis was conducted. The total number of meaningful concepts linked to categories in the ICF components (activities and participation; body functions; body structures; environmental factors) and the number of meaningful concepts which could not be linked (coded as not defined, not covered, health condition) were counted for each instrument. In the analyses the third- and fourth-level categories were rounded up and reported as second-level ICF categories. The percentage of agreement between the two coders was calculated for the first- and second-level ICF categories and codes initially selected for the meaningful concepts in each instrument and did not consider any revisions made by the third coder.

Second, the content of each instrument was examined. Since there is no consensus on how to operationalize participation, for the content analysis participation was defined broadly and included all domains within the activities and participation component. The content in each of the instruments was examined by reporting the: 1) coverage of the ICF chapters (domains) within the activities and participation component; 2) relevance of the meaningful concepts to the activities and participation component; and 3) context in which the activities and participation component categories are evaluated. Coverage was examined by calculating the number of activities and participation component domains included in each instrument and the percentage of questions containing ICF categories from the activities and participation component. Relevance was examined by determining if all the questions contain a meaningful concept linked to the activities and participation component (d-category). Since an instrument may contain meaningful concept(s) related to participation but an ICF category could not be selected, meaningful concepts coded as 'not defined' and 'not covered' were reviewed by one of the coders to determine if the meaningful concepts were similar to the content included in the activities and participation domains *d1 Learning and applying knowledge *through to *d9 Community, social and civic life*. Finally, to determine the context in which the activities and participation categories were evaluated, the percentage of questions containing ICF categories from the ICF components (body functions, body structures, environmental factors, personal factors) as well as those coded as 'health conditions' and 'not defined/not covered' were reported.

## Results

### Identification of the Participation Instruments

A review of the literature in September 2007 identified 3087 articles. After reviewing the articles based on the two stage eligibility process ten instruments were included: Impact on Participation Autonomy (IPA) [15,16], Keele Assessment of Participation (KAP) [17], PAR-PRO [18], Participation Measure-Post Acute Care (PM-PAC) [19], Participation Objective Participation Subjective (POPS) [20], Participation Scale (P-Scale) [21], Participation Survey/Mobility (PARTS/M) [22], Perceived Impact of Problem Profile (PIPP) [23], Rating of Perceived Participation (ROPP) [24], and World Health Organization Disability Assessment Schedule II (WHODAS II) [25]. The Participation Measure-Post Acute Care-Computerized Adaptive Test version (PM-PAC-CAT) [26] was added when the systematic search was updated in March 2008. For eight of the instruments (IPA, KAP, PARTS/M, PM-PAC, POPS, P-Scale, ROPP, WHODAS II) a copy of the instrument was available and so these instruments were included in the content analysis.

### Linking the Meaningful Concepts to the ICF

A total of 1351 meaningful concepts were identified in the eight instruments. In the P-Scale there are a total of 36 questions, however only 18 questions were assessed in this study since the meaningful concepts are not explicitly stated in 18 questions which ask 'how big a problem is it to you?' and follows the first question. In addition, there was no impact on the results by only including 18 questions from the P-Scale. The percentage of observed agreement between the two coders ranged between 91% (P-Scale) to 100% (ROPP) for the first-level ICF categories and codes and 77% (P-Scale) to 95% (ROPP) for the second-level ICF categories and codes. Level of agreement could not be reported for the IPA since this instrument was linked to the ICF classification using a similar methodology by the same coders in a previous study but coder agreement was not assessed.

The PARTS/M has the highest number of meaningful concepts (n = 545). Sixty nine percent (933/1351) of the meaningful concepts were linked to categories in the component activities and participation (see Table 2). No meaningful concepts were linked to personal factors. The categories from the activities and participation component that were coded based on the meaningful concepts are included as an Additional file (see Additional file 1: ICF categories in the component activities and participation based on the meaningful concepts). All of the instruments contain meaningful concepts linked to categories in the following activities and participation domains: *d4 Mobility*, *d6 Domestic life*, *d7 Interpersonal interactions and relationships*, *d8 Major life areas *and *d9 Community, social and civic life*. The categories within the ICF components body functions (b-categories) and environmental factors (e-categories) coded based on the meaningful concepts are included as an Additional file (see Additional file 2: ICF categories in the components body functions and environmental factors based on the meaningful concepts). Since the number of questions in each instrument varies, the number of questions (as well as a percentage of the total number of questions) that contain meaningful concepts linked to categories in the ICF components as well as the codes for meaningful concepts that could not be linked were calculated [see Additional file 3: Number of questions with ICF categories and codes (%)]. A summary of the results based on the criteria used to examine the instrument content is described in Table 3.

**Table 2:** Summary of the data abstracted from the participation instruments

		IPA	KAP	PARTS/M	PM-PAC	POPS	P-Scale	ROPP	WHODAS II
Number of meaningful concepts linked to ICF categories		122	49	479	117	144	47	153	42
Body function				40			1		3
Activity/Participation		56	27	379	103	135	42	153	38
Environmental factors		66	22	60	14	9	4		1
Number of meaningful concepts not linked to ICF categories		84		66	9				39
Health conditions		82		40	2				36
Not defined or not covered		2		26	7				3

Abbreviations:

IPA, Impact on Participation and Autonomy; KAP, Keele Assessment of Participation; PARTS/M, Participation Survey/Mobility; PM-PAC, Participation Measure-Post Acute Care; POPS, Participation Objective Participation Subjective; P-Scale, Participation Scale; ROPP, Rating of Perceived Participation; WHODAS II, World Health Organization Disability Assessment Schedule II

**Table 3:** Summary of the criteria used to assess the content of the participation instruments

Instrument	**Criteria #1**:	**Criteria #2**:	**Criteria #3**:
	Activities and participation domains* covered	All questions contain categories in the ICF activities and participation component	Questions contain meaningful concepts related to: body functions; body structures; environmental factors; personal factors; health condition
IPA	d4 to d9	yes	environmental factors; health condition
KAP	d3 to d9	yes	environmental factors
PARTS/M	d4 to d9	yes	body functions; environmental factors; health condition
PM-PAC	d3 to d9	yes†	environmental factors; health condition
POPS	d3, d4, d6 to d9	yes	environmental factors
P-Scale	d1, d3 to d9	no	body functions; environmental factors
ROPP	d3 to d9	yes	none
WHODAS II	d1, d3 to d9	no†	body functions; environmental factors; health condition

Abbreviations:

IPA, Impact on Participation and Autonomy; KAP, Keele Assessment of Participation; PARTS/M, Participation Survey/Mobility; PM-PAC, Participation Measure-Post Acute Care; POPS, Participation Objective Participation Subjective; P-Scale, Participation Scale; ROPP, Rating of Perceived Participation; WHODAS II, World Health Organization Disability Assessment Schedule II

Notes:

* d1 Learning and applying knowledge; d2 General tasks and demands; d3 Communication; d4 Mobility; d5 Self-care; d6 Domestic life; d7 Interpersonal interactions and relationships; d8 Major life areas; d9 Community, social and civic life

† Contains 'not defined' or 'not covered' codes that are considered to be similar in content to the domains d1 to d9 in the activities and participation component.

#### Overview of the Content in the Participation Instruments

##### Impact on Participation and Autonomy (IPA)

The IPA contains 41 questions and 206 meaningful concepts. The activities and participation domains *d6 Domestic life*, *d7 Interpersonal interactions and relationships*, *d8 Major life areas *have the most coverage, with 22% of questions (n = 9 questions) covering each domain. In the IPA many questions ask the respondent to consider the use of assistance or the use of aids and these meaningful concepts were linked to categories in the environmental factor domains *e3 Support and relationships *and *e1 Products and technology, respectively*. There were 84 meaningful concepts in the IPA which could not be linked to the ICF. The instructions in the IPA ask the respondent to consider all the questions in the context of their 'health' or 'disability' and both of these were considered meaningful concepts and were linked to 'health conditions'. The meaningful concept coded as 'not covered' was 'living life' and the concept considered 'not defined' was 'personal life', which is stated in the preface to this question. All the questions in the IPA have at least one meaningful concept related to *d4 Mobility *through to *d9 Community, social and civic life*.

##### Keele Assessment of Participation (KAP)

The KAP instrument contains a total of 15 questions, including the screening questions, and 49 meaningful concepts were linked to the ICF classification. Meaningful concepts were linked to *d3 Communication *through to *d9 Community, social and civic life*. The activities and participation domains *d6 Domestic life *and *d8 Major life areas *have the greatest coverage, with 27% (n = 4 questions) and 33% (n = 5 questions) of questions covering each domain, respectively. The instructions in the KAP tell the respondent to consider the 'use of assistance' or the 'use of products and technology' and e-categories for these meaningful concepts were identified and linked. All of the meaningful concepts were linked to ICF categories and each question contains an ICF category from *d3 Communication *through to *d9 Community, social and civic life*.

##### Participation Measure-Post Acute Care (PM-PAC)

The PM-PAC instrument contains 51 questions. One hundred and twenty six meaningful concepts were identified and 117 of these were linked to the ICF. The PM-PAC has two questions which ask about 'filing your taxes' and 'completing forms for insurance or disability benefits' where the instructions ask the respondent to consider any assistance (*e3 Support and relationships*) or services (*e5 Services, systems and policies*) available to them. There are also meaningful concepts which were coded as 'not defined', for example 'other activities' and 'days away from your home'. Although the PM-PAC has questions which do not contain any ICF categories from domains in the activities and participation component, there is at least one meaningful concept in each question related to these domains. Examples of meaningful concepts which were coded as 'not defined' or 'not covered' but considered related to the concept of participation include 'days away from your home', 'accomplishing tasks', 'filing taxes' and 'completing forms for insurance or disability benefits'.

##### Participation Objective Participation Subjective (POPS)

The POPS contains 78 questions and all of the 144 meaningful concepts identified could be linked to the ICF classification. The meaningful concepts primarily cover the domains *d6 Domestic life *through *d9 Community, social and civic life*. Six meaningful concepts were linked to *d350 Conservation *in the domain *d3 Communication *and the meaningful concepts in *d4 Mobility *are all related to transportation (*d470 Using transportation *and *d475 Driving*). All of the questions contain meaningful concepts linked to domains in the activities and participation component. The meaningful concept 'using a phone' was identified in nine questions asking about socialization and coded as an environmental factor (*e125 Products and technology for communication*). Neither the instructions nor the questions asked the respondent to consider his or her health condition when considering aspects of participation.

##### Participation Scale (P-Scale)

The P-Scale contains 36 questions, however, in this study only 18 questions were considered since the meaningful concepts are not explicitly stated in 18 questions which ask 'how big a problem is it to you?'. A total of 47 meaningful concepts were identified and all the concepts were linked to the ICF classification. The meaningful concepts cover all of the activities and participation domains with the exception of *d2 General tasks and demands*. One meaningful concept, 'confidence' was linked to body functions (*b126 Temperament and personality functions*). There are three questions with meaningful concepts asking about attitudes (*e4 Attitudes*). The P-Scale has one question, 'In your home, are the eating utensils you use kept with those used by the rest of the household?', where the meaningful concepts are only related to environmental factors; the meaningful concepts 'eating utensils' and 'attitudes of family members' were linked to the ICF categories '*e115 Products and technology for personal use in daily living*' and '*e410 Individual attitudes of immediate family members*'. This question seems to ask about the observable consequences of others' attitudes and so it was not considered to be related to the concept of participation. It is the only question which did not have a meaningful concept related to the domains in the activities and participation component. None of the questions include meaningful concepts related to 'health condition'

##### Participation Survey/Mobility (PARTS/M)

The PARTS/M has a total of 159 questions, including screening questions. There are a total of 545 meaningful concepts and 479 of these could be linked to the ICF classification. Meaningful concepts in the PARTS/M were linked to ICF categories in *d3 Communication *through to *d9 Community, social and civic life *and each question had a minimum of one d-category from these ICF domains. In the PARTS/M, for each of the 20 aspects of participation included there is a question which asks if either 'pain' (*b280 Sensation of pain*) or 'fatigue' (*b4552 Fatiguability*) limits participation. There are also questions which ask about the use of 'assistance', 'adaptations' or 'special equipment' and these meaningful concepts were linked to e-categories within the ICF component environmental factors. Meaningful concepts which could not be linked to the ICF included concepts such as 'use of accommodations' and 'physical impairment' and were each coded as 'not defined' and 'health condition', respectively.

##### Rating of Perceived Participation (ROPP)

The ROPP contains 69 questions and 153 meaningful concepts. All of the meaningful concepts were linked to *d3 Communication *through to *d9 Community, social and civic life *and each question contains a minimum of at least one meaningful concept from these domains. Categories in the domain *d8 Major life areas *have the most coverage, with 22% of questions (n = 15 questions) containing ICF categories from this domain. There were no meaningful concepts linked to the ICF components body functions/structures or environmental factors and all of the meaningful concepts could be linked.

##### World Health Organization Disability Assessment Schedule II (WHODAS II)

The WHODAS II contains 36 questions and a total of 81 meaningful concepts. Forty-two meaningful concepts were linked to the ICF classification. The meaningful concepts covered all of the activities and participation domains with the exception of *d2 General tasks and demands*. Meaningful concepts were also linked to body functions as well as environmental factors. In terms of body functions, three questions which ask about 'remembering to do important things', 'being emotionally affected' and 'living with dignity', were linked to *b144 Memory functions*, *b152 Emotional functions *and *b1Mental functions*, respectively. There were 39 meaningful concepts which could not be linked to the ICF classification. Instructions in the WHODAS II state the respondent should consider his or her health for each question, resulting in 36 'health condition' codes. Three meaningful concepts were considered to be 'not defined' ('staying by yourself for a few days') or 'not covered' ('impact on your family'). In the WHODAS II there are five questions which do not contain any categories in the activities and participation domains and were also not considered to be related to participation; these questions include meaningful concepts related to body functions (*b1 Mental functions, b144 Memory functions, b152 Emotional functions*), 'not covered' ('impact on your family') or 'not defined' ('barriers or hindrances in the world around you').

## Discussion

### Concept of Participation

By linking the meaningful concepts identified in the participation instruments, it was possible to determine which ICF categories the instruments include. In this study an instrument was considered to assess the concept of participation and included if its domains cover a minimum of three chapters (domains) between *d3 Communication *and *d9 Community, social and civic life *in the ICF component activities and participation. This broad definition of participation was used since there is no consensus regarding how activity is differentiated from participation [2,5,11-14] and selecting chapter headings provided objective criteria. In considering which activities and participation domains the instruments cover, an even broader definition of participation was used by also including *d1 Learning and applying knowledge *and *d2 General tasks and demands *since these domains may have been considered relevant to the concept of participation by the instrument developers. Perenboom and Chorus [2], however, considered a question to be assessing participation if it asks about "actual or perceived participation (involvement, autonomy, social role)" (page 578) and so different results would be obtained using this definition.

### Content of the Participation Instruments

Although all the instruments cover six to eight of the nine activities and participation domains, there are differences in the actual content. All of the instruments include content from domains *d6 Domestic life*, *d7 Interpersonal interactions and relationships*, *d8 Major life areas *and *d9 Community, social and civic life*. However, there are differences in whether the domains *d3 Communication*, *d5 Self-care *and certain aspects of *d4 Mobility *are considered aspects of participation.

Four instruments (PM-PAC, P-Scale, ROPP, WHODAS II) intend to assess *d3 Communication *based on the materials describing their development and ICF categories from *d3 Communication *were noted for all these instruments. Meaningful concepts linked to categories in *d3 Communication *were also identified in the KAP and POPS which is likely not the major focus, as the questions have meaningful concepts linked to multiple ICF domains. For example, in the POPS the question 'How many times do you speak with your neighbour?' includes the meaningful concept 'conversation' which was coded as *d350 Conversation *but it is only a minor meaningful concept and the major meaningful concept is 'relationship with neighbour(s)', coded as *d7501 Informal relationships with neighbours*. In some instruments, such as the PM-PAC, assessing communication is a major focus ('How much are you limited in watching or listening to the television or radio?'). Empirical findings suggest that it is difficult to demonstrate discriminant validity among participation domains [15,17] and this may be a result of overlapping content. In future studies it may be beneficial to identify and code the major and minor meaningful concepts, since this could assist with developing a priori hypotheses regarding expected correlations between instrument domains.

All of the instruments contain meaningful concepts linked to categories in *d5 Self-care *with the exception of the POPS. When the POPS was developed self-care was not included since participation was operationalized as "engagement in activities that are intrinsically social, that are part of household or other occupational role functioning, or that are recreational activities occurring in community settings" (page 463) and self-care did not qualify [20]. The PM-PAC does not intend to assess self-care [19] but there were two meaningful concepts linked to *d5 Self-care*. One question in the PM-PAC asks about 'exercising' which was coded as *d5701Managing diet and fitness *and the other question asks about 'providing self-care to yourself', which was coded as *d5 Self-care*. In terms of mobility, all of the instruments contain meaningful concepts linked to categories in *d4 Mobility *and all the instruments intend to include content from this domain. Three instruments (IPA, PARTS/M, WHODAS II) operationalize moving in the home using specific phrases such as 'getting out of bed', 'getting out of a chair' (PARTS/M) or 'getting up and going to bed' (IPA). In the other instruments, mobility includes broader statements such as 'moving or getting around the home' (KAP, PM-PAC, P-Scale, ROPP) and in the POPS mobility includes only using transportation.

Two instruments, the P-Scale and WHODAS II, were considered to have content not related to the concept of participation, which was defined broadly as ICF categories in the activities and participation domains *d1 Learning and applying knowledge *to *d9 Community, social and civic life*. The P-Scale has one question which only asks about the observable attitudes of others ('In your home, are the eating utensils you use kept with those used by the rest of the household?'). The WHODAS II contains five questions which ask about content related to body functions (e.g. 'remembering' which was linked to *b144 Memory functions*) or were not covered/not defined (e.g. 'barriers or hindrances in the world around you'). By linking the meaningful concepts to the ICF classification it was evident that not all questions appear to assess participation as defined in the ICF. This information may assist users in understanding what the questions assess and aid in selecting an instrument depending on his or her purpose, since this may or may not be an issue.

### Linking the Meaningful Concepts to the ICF

The methodology published by Cieza et al. [7] was used to identify and link meaningful concepts to the ICF. Our results for the activities and participation codes for the WHODAS II can be compared to a study by Cieza and Stucki [10], which also linked the WHODAS II to the ICF. It is difficult to compare the results from these two studies directly since Cieza and Stucki [10] used an older version of the linking rules [7] and we modified the linking rules by including 'health condition' as a meaningful concept if it was included in the instructions. Cieza and Stucki [10] identified 38 meaningful concepts and in our study we had 45 not including coding 'health condition', however, we did not include the five questions in the WHODAS II on general health and it appears that Cieza and Stucki [10] did. Both studies had the same number of meaningful concepts linked to body functions (n = 3), environmental factors (n = 1) and 'not defined' (n = 2). There were some differences. We linked 38 meaningful concepts to categories from activities and participation and Cieza and Stucki [10] linked 30 meaningful concepts and we linked one meaningful concept to 'not covered' whereas these authors linked two meaningful concepts.

The implications of not reliably determining if the meaningful concepts can be linked to the ICF classification or differences in the ICF categories and codes selected can impact the results and how the questions in the instruments are interpreted. It has been recognized that there are a number of challenges with using the linking rules (e.g. establishing the meaningful concepts contained in the assessment items) [27]. Offering on-line training on how to use the ICF linking rules and presenting difficult coding examples are types of initiatives that could help improve the standardization of this methodology.

### Participation and Other ICF Categories and Codes

Meaningful concepts included in the instructions as well as within each question were examined to determine the context in which aspects of participation are assessed. The ICF states that disability is a dynamic process which results from the interaction of the ICF components (body structures, body functions, activities and participation) and the contextual factors (environment, personal factors) [3]. It is helpful to identify what is asked in relation to participation; for example, for every participation topic area (e.g. dressing, working inside the home) included in the PARTS/M, a question is asked if participation is impacted by pain and/or fatigue. Clinically it is useful to determine the impact of factors such as pain and fatigue, since similar to environmental factors they can be potentially modified in order to enhance participation.

As stated by Nordenfelt [13] and others [28], activity and participation must occur in an environment. In the ICF there is reference to a 'standard environment' versus 'usual environment' and this distinction is one way activity is differentiated from participation [3]. It is interesting how environmental factors asking about assistance or equipment are included in some instruments (IPA, KAP, PARTS/M, PM-PAC, POPS, P-Scale) but not in other instruments (ROPP, WHODAS II). The PARTS/M specifically assesses the use of assistance and the frequency which accommodations, adaptations or special equipment is used. Asking about the use of equipment and assistance is important clinically since a person's environment can often be modified to enhance their participation. Further qualitative and quantitative studies will determine if respondents inherently consider their environment when answering the questions.

Similar to environmental factors, there is variation in whether a participation restriction is attributed to a health condition. In the WHODAS II and IPA the instructions state that the respondent should consider their health condition or disability. In the PARTS/M there are specific questions which ask if the person's participation is limited by their illness or physical impairment. Dubuc et al. [29] demonstrated the importance of specifying whether the participation restriction is a result of a health condition or not, especially for areas which are highly influenced by environmental factors. By asking if the participation restriction is a result of a health condition, it underestimated the influence of the environment since subjects focussed on the implications due their health and did not often consider the restrictions in the physical and social environment [29]. More research should determine the best way to assess these influencing factors. The PARTS/M offers the advantage of asking specific questions with and without the influence of health and the environment which may help determine the causes of the participation restrictions and also provide potentially 'pure measures' of participation. None of the instruments have meaningful concepts coded as personal factors, which is not surprising since this data is often collected separately (e.g. age, gender) in research studies. Further studies should compare questions that either attribute or do not attribute participation to factors such as the environment or health conditions to determine if these phrases influence a person's response.

### Study Limitations

There are several limitations to this study which need to be considered when interpreting the results. In this study only instruments which were developed using the ICF were included and the meaningful concepts were linked to the ICF classification, which limits the findings to how participation is conceptualized in the ICF. In addition, the criteria assume it is desirable to have an instrument cover the majority of areas within a multidimensional concept such as participation and so it may not be suitable for instruments which focus on selected areas such as employment. By linking the meaningful concepts in the questions to the ICF classification it provided an objective evaluation, however, it is possible that we did not capture the correct meaning of the questions. Since very few studies have linked the instruments used in this study to the ICF classification, the results from this study should be confirmed in other studies. Interpreting the questions and determining the meaningful concepts can be influenced by culture and the experience of the coders and enhancements to the ICF linking rules will help improve the assessment of content validity in these types of studies.

## Conclusion

In summary, this study linked eight instruments measuring participation to the ICF. The benefits of linking content from instruments to the ICF have been described in various studies [9,10,30]. These benefits include enabling users to review the content as part of the selection process, providing a standardized approach to comparing the content and informing future revisions of existing instruments. An enhancement to the linking methodology used in this study enabled the role of contextual factors as well as attribution of the participation restriction due to health to be further examined within each question. Including contextual factors in the ICF is an important step forward and empirical research comparing results from instruments which either include and or do not include contextual factors will further advance the measurement of participation. The instruments all contain content from the domains *d6 Domestic life *to *d9 Community, social and civic life *but there is variability in whether content from domains *d1 Learning and applying knowledge*, *d3 Communication *and *d5 Self-care *is included. Two instruments, P-Scale and WHODAS II have questions which did not contain any ICF categories related to the domains in the activities and participation component, which suggest these questions may not measure aspects of participation. The differences in content, attributing participation restrictions to health and asking about aspects of the environment should be considered when selecting a participation instrument as it may or may not be desirable depending on the intended purpose.

## Competing interests

The authors declare that they have no competing interests.

## Authors' contributions

VKN conceived the idea, conducted the literature review, was primarily involved with the data coding, analyzed and assisted in the interpretation of the results and wrote the manuscript. JAK, LN conceived the idea, provided guidance on the data coding, assisted in the interpretation of the results and commented on the manuscript. AC assisted with the data coding and assisted in interpreting the results. JS, LCM and MFD were involved in the interpretation of the results and commented on the manuscript. All authors read and approved the final manuscript.

## Appendix

### List of search terms

#### Conceptual model terms

▪ International Classification of Functioning, Disability and Health (ICF)

▪ International Classification of Impairment, Disability and Handicap (ICIDH)

▪ ICIDH-2

▪ World Health Organization

#### Participation related terms

▪ participation

▪ handicap

▪ patient participation

▪ consumer participation

▪ community re-integration

▪ community integration

▪ social adaptation

▪ social adjustment

▪ independent living

▪ daily life activity

▪ instrumental activities of daily living

▪ quality of life

#### Instrument terms

▪ questionnaire

▪ instrument

▪ instrument evaluation

▪ health survey

▪ health assessment questionnaire

▪ psychometrics

▪ disability evaluation

▪ outcome assessment

▪ rehabilitation

## Supplementary Material

Additional file 1 **ICF categories in the component activities and participation based on the meaningful concepts**. The data include a detailed listing of the ICF categories from the activities and participation component coded based on the meaningful concepts. Click here for file

Additional file 2 **ICF categories in the components body functions and environmental factors based on the meaningful concepts**. The data include a detailed listing of the ICF categories from the components body functions and environmental factors coded based on the meaningful concepts. Click here for file

Additional file 3 Number of questions with ICF categories and codes (%). The data include the number of questions (and the percentage of the total number of questions) that contain meaningful concepts linked to ICF categories within the ICF components as well as the codes for meaningful concepts which could not be linked. Click here for file
