# Supplementary material for: Comparing the content of participation instruments using the International Classification of Functioning, Disability and Health
Source: Health Qual Life Outcomes. 2009 Nov 13;7:93. doi: 10.1186/1477-7525-7-93 (PMC2785762; doi:10.1186/1477-7525-7-93)
Supplement: Additional file 1 — ICF categories in the component activities and participation based on the meaningful concepts. The data include a detailed listing of the ICF categories from the activities and participation component coded based on the meaningful concepts. [file 1477-7525-7-93-S1.doc]

**Additional File 1: ICF categories in the component activities and participation based on the meaningful concepts**

ICF category IPA KAP PARTS/M PM-PAC POPS P-Scale ROPP WHODAS II

**d1 Learning and applying**

**knowledge** 1

d155 Acquiring skills 2

d160 Focusing attention 1

d175 Solving problems 1

**d2 General tasks and**

**demands**

**d3 Communication** 1 4 1

d310 Communicating with-receiving-spoken messages 2 1

d325 Communicating with-receiving-written messages 3

d330 Speaking 3

d335 Producing nonverbal messages 12

d345 Writing messages 1 3

d350 Conversation 6 2

d355 Discussion 1

d360 Using communication devices and techniques 4 12

**d4 Mobility** 1 4 10

d410 Changing basic body position 1 10 1

d415 Maintaining a body position 1

d450 Walking 1

d455 Moving around

d460 Moving around in different locations 2 2 43 7 10 6 2

d470 Using transportation 12 9

d475 Driving 3

**Additional File 1: ICF categories in the component activities and participation based on the meaningful concepts**

ICF category IPA KAP PARTS/M PM-PAC POPS P-Scale ROPP WHODAS II

**d5 Self-care** 1 1 1 2 7

d510 Washing oneself 2 1 18 1

d520 Caring for body parts

d530 Toileting 1 1 18

d540 Dressing 2 1 18 6 1

d550 Eating 1 1 5 3 1

d560 Drinking 1 3

d570 Looking after one’s health 1 1 2

**d6 Domestic life** 3 1 4 2

d620 Acquisition of goods and services 2 9 3

d630 Preparing meals 1 3 6 3

d640 Doing housework 4 21 1 3 1 6 2

d650 Caring for household objects 2 1 21 1 6 3

d660 Assisting others 2 4 2 6 4 4

**d7 Interpersonal interactions**

**and relationships** 5 1 1 1

d720 Complex interpersonal interactions 3 1

d730 Relating with strangers 3 1

d740 Formal relationships 1 1 3

d750 Informal social relationships 2 7 12 12 7 2

d760 Family relationships 1 23 8 6 1 6

d770 Intimate relationships 1 35 3 3 1

**d8 Major life areas** 4 4 7 3 6 5

d820 School education 1

d825 Vocational training 2 3

**Additional File 1: ICF categories in the component activities and participation based on the meaningful concepts**

ICF category IPA KAP PARTS/M PM-PAC POPS P-Scale ROPP WHODAS II

**d8 Major life areas continued**

d830 Higher education 3

d840 Apprenticeship 1 3

d845 Acquiring, keeping and terminating a job 7 4 4 3 3

d850 Remunerative employment 1 2 9 3 1

d855 Non-remunerative employment 1 2 9 3

d860 Basic economic transactions 7 1 3

d865 Complex economic transactions 28 6 3

d870 Economic self-sufficiency 2 1 3 1 3 1

**d9 Community, social**

**and civic life** 2 2 6 2 1

d910 Community life 2 3 5 3

d920 Recreation and leisure 5 62 19 24 4 6 1

d930 Religion and spirituality 14 3 6 2 3 1

d950 Political life and citizenship 28 3 4

Abbreviations:

ICF, International Classification of Functioning, Disability and Health; IPA, Impact on Participation and Autonomy; KAP, Keele Assessment of Participation; PARTS/M, Participation Survey/Mobility; PM-PAC, Participation Measure-Post Acute Care; POPS, Participation Objective Participation Subjective; P-Scale, Participation Scale; ROPP, Rating of Perceived Participation; WHODAS II, World Health Organization Disability Assessment Schedule II
