# Supplementary material for: Comparing the content of participation instruments using the International Classification of Functioning, Disability and Health
Source: Health Qual Life Outcomes. 2009 Nov 13;7:93. doi: 10.1186/1477-7525-7-93 (PMC2785762; doi:10.1186/1477-7525-7-93)
Supplement: Additional file 2 — ICF categories in the components body functions and environmental factors based on the meaningful concepts. The data include a detailed listing of the ICF categories from the components body functions and environmental factors coded based on the meaningful concepts. [file 1477-7525-7-93-S2.doc]

**Additional File 2: ICF categories in the components body functions and environmental factors based on the meaningful concepts**

ICF category IPA KAP PARTS/M PM-PAC POPS P-Scale ROPP WHODAS II

***Body Functions (b)***

**b1 Mental functions** 1

b126 Temperament and personality functions 1

b144 Memory functions 1

b152 Emotional functions 1

**b2 Sensory functions**

b280 Sensation of pain 20

**b4 Functions of the cardiovascular, hameatological,**

**immunological and respiratory systems**

b455 Exercise tolerance functions 20

***Environmental Factors (e)***

**e1 Products and technology** 2 1 24

e115 Products and technology for personal use in

daily living 15 5 8 1

e120 Products and technology for personal indoor

and outdoor mobility and transportation 5 2 3 1

e125 Products and technology for communication 1 1 9

e130 Products and technology for education 2 1

e135 Products and technology for employment 5 1 2

e140 Products and technology for culture, recreation

and sport 2 2

e145 Products and technology for the practice of

religion and spirituality 1

**Additional File 2: ICF categories in the components body functions and environmental factors based on the meaningful concepts**

ICF category IPA KAP PARTS/M PM-PAC POPS P-Scale ROPP WHODAS II

**e3 Support and relationships** 32 11 20 7

**e4 Attitudes** 1 1

e410 Individual attitudes of immediate family

members 2

e425 Individual attitudes of acquaintances, peers,

colleagues, neighbours and community members 1 1

**e5 Services, systems and policies** 3

e575 General social support services, systems and policies 3

Abbreviations:

ICF, International Classification of Functioning, Disability and Health; IPA, Impact on Participation and Autonomy; KAP, Keele Assessment of Participation; PARTS/M, Participation Survey/Mobility; PM-PAC, Participation Measure-Post Acute Care; POPS, Participation Objective Participation Subjective; P-Scale, Participation Scale; ROPP, Rating of Perceived Participation; WHODAS II, World Health Organization Disability Assessment Schedule II
