# Supplementary material for: Comparing the content of participation instruments using the International Classification of Functioning, Disability and Health
Source: Health Qual Life Outcomes. 2009 Nov 13;7:93. doi: 10.1186/1477-7525-7-93 (PMC2785762; doi:10.1186/1477-7525-7-93)
Supplement: Additional file 3 — Number of questions with ICF categories and codes (%). The data include the number of questions (and the percentage of the total number of questions) that contain meaningful concepts linked to ICF categories within the ICF components as well as the codes for meaningful concepts which could not be linked. [file 1477-7525-7-93-S3.doc]

**Additional File 3: Number of questions with ICF categories and codes (%)***

IPA KAP PARTS/M PM-PAC POPS P-Scale ROPP WHODAS II

Number of questions in the instrument 41 15 159 51 78 18† 69 36

***d1 Learning and applying knowledge***

# questions containing categories from d1 (%) 0 (0) 0 (0) 0 (0) 0 (0) 0 (0) 1 (6) 0 (0) 3 (8)

***d2 General tasks and demands***

# questions containing categories from d2 (%) 0 (0) 0 (0) 0 (0) 0 (0) 0 (0) 0 (0) 0 (0) 0 (0)

***d3 Communication***

# questions containing categories from d3 (%) 0 (0) 1 (7) 0 (0) 7 (14) 6 (8) 1 (6) 13 (19) 2 (6)

***d4 Mobility***

# questions containing categories from d4 (%) 4 (10) 2 (13) 19 (12) 8 (16) 6 (8) 3 (17) 13 (19) 5 (14)

***d5 Self-care***

# questions containing categories from d5 (%) 5 (12) 1 (7) 29 (18) 2 (4) 0 (0) 1 (6) 10 (14) 3 (8)

***d6 Domestic life***

# questions containing categories from d6 (%) 9 (22) 4 (27) 17 (11) 4 (8) 18 (23) 2 (11) 7 (10) 4 (11)

***d7 Interpersonal interactions and relationships***

# questions containing categories from d7 (%) 9 (22) 1 (7) 22 (14) 11 (22) 24 (31) 3 (17) 13 (19) 5 (14)

***d8 Major life areas***

# questions containing categories from d8 (%) 9 (22) 5 (33) 25 (16) 14 (27) 12 (15) 3 (17) 15 (22) 5 (14)

***d9 Community, social and civic life***

# questions containing categories from d9 (%) 7 (17) 2 (13) 58 (36) 11 (22) 30 (38) 4 (22) 7 (10) 2 (6)

**Additional File 3: Number of questions with ICF categories and codes (%)***

IPA KAP PARTS/M PM-PAC POPS P-Scale ROPP WHODAS II

***Body Functions***

# questions containing categories from

body functions (b-categories) (%) 0 (0) 0 (0) 20 (13) 0 (0) 0 (0) 1 (6) 0 (0) 3 (8)

***Environmental Factors***

# questions containing categories from

environmental factors (e-categories) (%) 34 (83) 11 (73) 40 (25) 7 (14) 9 (12) 3 (17) 0 (0) 1 (3)

***Health Conditions***

# questions containing meaningful concepts

coded as health conditions (%) 41 (100) 0 (0) 20 (13) 1 (2) 0 (0) 0 (0) 0 (0) 36 (100)

***Not Defined/Not Covered***

# questions containing meaningful concepts

coded as not defined/ not covered (%) 1 (2) 0 (0) 26 (16) 7 (14) 0 (0) 0 (0) 0 (0) 3 (8)

Abbreviations:

ICF, International Classification of Functioning, Disability and Health; IPA, Impact on Participation and Autonomy; KAP, Keele Assessment of Participation; PARTS/M, Participation Survey/Mobility; PM-PAC, Participation Measure-Post Acute Care; POPS, Participation Objective Participation Subjective; P-Scale, Participation Scale; ROPP, Rating of Perceived Participation; WHODAS II, World Health Organization Disability Assessment Schedule II

Note:

* % is the number of questions with ICF categories or codes/total number of questions

† The P-Scale has 36 questions but only 18 questions are considered since the meaningful concepts are not explicitly stated in 18 questions which ask ‘how big a problem is it to you?’ for each question.
